# A systematic review of guidelines on screening for celiac disease in children with thyroid disease and vice versa

**DOI:** 10.3389/fped.2025.1538409

**Published:** 2025-03-31

**Authors:** Talia D’Ambrosio, Silvia Bianchin, Roberto Gastaldi, Noemi Zampatti, Valentina Biagioli, Alessandro Naim, Federica Malerba, Paolo Gandullia, Mohamad Maghnie, Marco Crocco

**Affiliations:** ^1^ Pediatric Clinic, University of Ferrara, Ferrara, Italy; ^2^ Department of Neuroscience, Rehabilitation, Ophthalmology, Genetics, Maternal and Child Health (DINOGMI), University of Genova, Genoa, Italy; ^3^ Pediatric Endocrinology Unit, Department of Pediatrics, IRCCS Istituto Giannina Gaslini, Genoa, Italy; ^4^ Pediatric Gastroenterology and Endoscopy Unit, IRCCS Istituto Giannina Gaslini, Genoa, Italy; ^5^ Department of Pediatrics, IRCCS Giannina Gaslini, Genova, Italy

**Keywords:** celiac disease, thyroid, hypothyroidism, hyperthyroidism, autoimmune thyroid disease, guideline, screening

## Abstract

**Introduction:**

Autoimmune thyroid diseases (ATD) are the most prevalent autoimmune disorders associated with celiac disease (CD). Both conditions can often be detected through serological screening in asymptomatic patients over several years. Various guidelines for screening thyroid disease (TD) are available in children with CD and vice versa.

**Methods:**

We conducted a systematic review to identify the most recent and relevant guidelines, comparing their recommendations to analyze key differences and suggesting a practical clinical approach.

**Results:**

Out of 1,294 articles reviewed, we identified 20 guidelines published between January 2013 and January 2024. These guidelines, primarily from gastroenterological organizations in Europe and North America, recommend different timings and methods for screening the co-occurrence of these diseases, both at diagnosis and during follow up. Some guidelines recommend only clinical follow-up without routine serological screening. There is limited consensus on screening for TD [using thyroid-stimulating hormone test (TSH)] in asymptomatic children newly diagnosed with CD, and even less agreement on screening for CD [using anti-transglutaminase antibodies (tTG) immunoglobulin A (IgA) test and total IgA] in children newly diagnosed with TD. No standardized procedures exist for managing patients with isolated low tTG and human leukocyte antigen (HLA) genotyping is rarely recommended as a first- line screening method.

**Discussion:**

Over the past decade, there has been a growing recognition of the importance of identifying children with co-occurrence of CD and TD who could benefit from early treatment, even in the absence of symptoms. However, international guidelines still show a lack of consensus regarding screening for these frequently associated autoimmune diseases, with notable differences in the use of HLA testing and follow-up protocols.

## 1 Introduction

Celiac disease (CD) is an autoimmune disorder triggered by gluten consumption in genetically predisposed individuals (1). Patients with CD have an increased risk of developing other autoimmune diseases including thyroid disease (TD), diabetes mellitus type 1 (T1DM), Addison's disease, skin disorders and multiple endocrine syndromes (2–4). Conversely, individuals with autoimmune thyroid disease (ATD) such as Hashimoto's thyroiditis (HT) and Graves' disease (GD) often present with CD (5). This frequent comorbidity may be attributed to a shared genetic susceptibility.

The clinical onset of CD or ATD can manifest through various signs and symptoms common to both conditions including growth retardation, constipation or diarrhea, irritability, behavioral changes, and reduced quality of life. Both diseases may remain asymptomatic for many years and are often identified only through serological screening (3, 6–10).

Optimizing the diagnostic approach is crucial in order to preserve the quality of life (11–13) and prevent the complications associated with delayed diagnosis (14, 15), while considering the costs and potential loss of quality of life from unnecessary medical testing.

Various gastroenterological and endocrinological societies have proposed different approaches to screening for thyroid disorders in children with CD and for CD in children with ATD.

To address this, we conducted a systematic review of the lates international guidelines from prominent pediatric endocrinological and gastroenterological societies. We compared their recommendations regarding the timing and types of screening tests for CD in children with TD and vice versa. Based on our findings, we propose a practical clinical flow chart to harmonize the management of these patients.

## 2 Material and methods

To identify the most recent national and international guidelines for CD and TD we performed a database search on PubMed selecting English language publications from the last decade (between January 2013 and January 2024) from leading gastroenterological and endocrinological pediatric societies. The following keywords were selected based on population (P) and Comparison (C) of PICO model: Coeliac Disease or Celiac Disease; Thyroid or Hypothyroidism or Hyperthyroidism or Autoimmune Thyroid disease; Guideline or Position Paper; Management through the following text strings: [“Celiac disease” (All Fields) OR “coeliac disease” (All Fields) OR “gluten-sensitive enteropathy” (All Fields) OR “thyroiditis” (All Fields) OR “hypothyroidism” (All Fields) OR “hyperthyroidism” (All Fields) OR “Graves’ disease” (All Fields) OR “Hashimoto thyroiditis” (All Fields) OR “thyroid disease” (All Fields)] AND [“guideline” (All Fields) OR “guidelines” (All Fields) OR “position paper” (All Fields) OR “guidance” (All Fields)] AND [“manage” (All Fields) OR “management” (All Fields) OR “follow-up” (All Fields) OR “disease management” (All Fields) OR “treatment” (All Fields)].

Based on the knowledge of all authors, we added guidelines from the Italian Society for Pediatric Endocrinology and Diabetology (ISPED/SIEDP) published in Italian.

The following information was extracted independently by two authors (TDA and NZ) using an extraction table (Microsoft® Excel® for Microsoft 365 MSO 64-bit, Microsoft Corporation Redmond, Washington, USA) including: the surname of the first author and publication date, country, timing and type of test screening at diagnosis or follow up, both for patients with CD and TD. Any discrepancies were resolved through discussion and by consulting a third author (MC).

The source of funding and conflicts of interest of authors were evaluated to assess the risk of bias within documents and in syntheses owing to missing results (16).

## 3 Results

**Figure 1:**
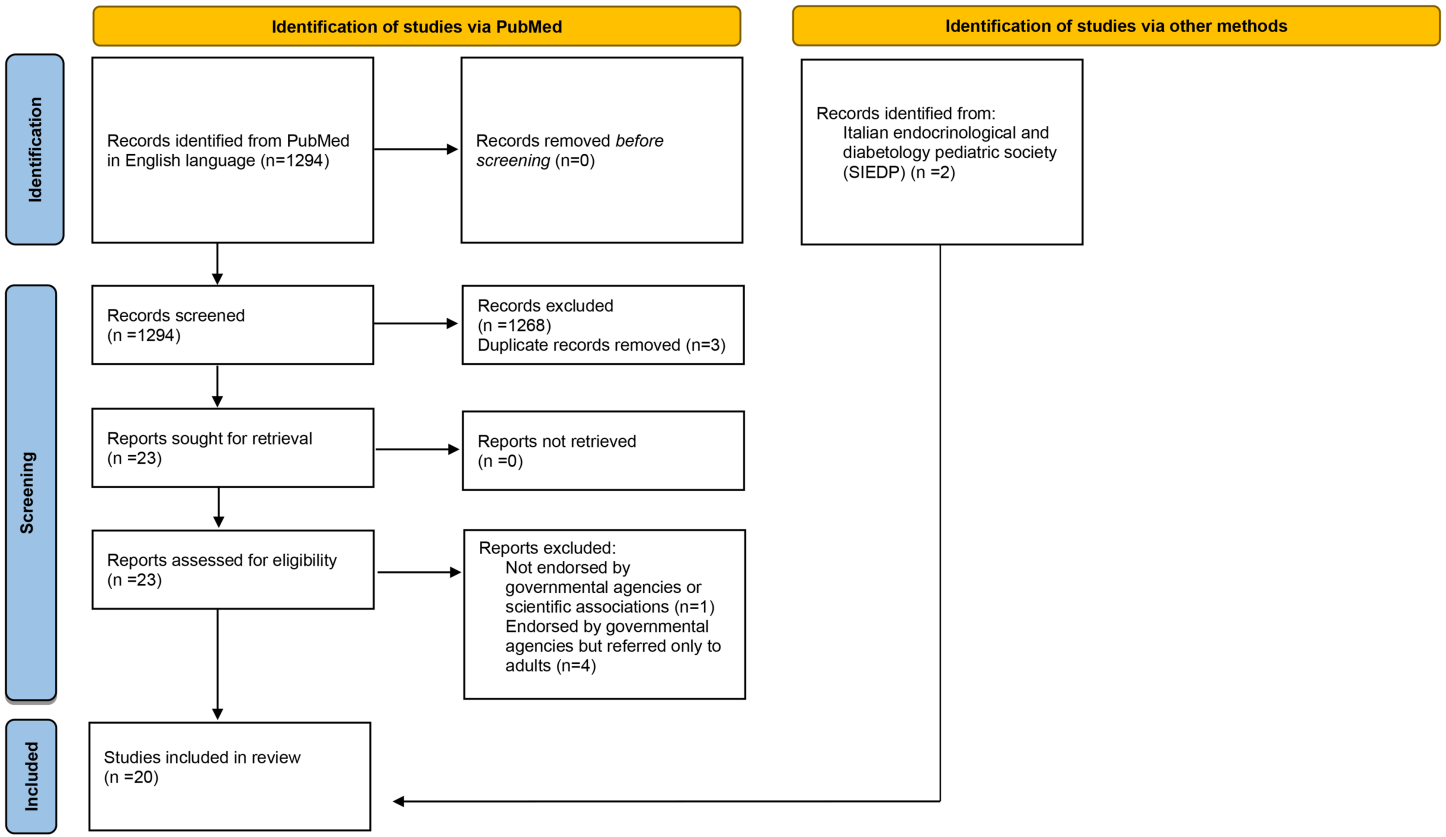
PRISMA flow diagram.

A total of 1,294 papers were identified by the search and a further 2 documents edited in Italian were added. Resulting in 20 documents included in the comparative analysis as reported in Figure 1.

We compared the recommendations emerging from different continents: twelve from Europe, four of which from the United Kingdom [BSPGHAN (17), NICE 2015 and 2020 (18, 19), BTA (20)], eight from the European Union [ESPGHAN 2020 and 2022 (21, 22), ESsCD (23), ETA 2014 and 2022 (24, 25), SIEDP (26, 27)], Italian societies of gastroenterology [SIGE-SIGENP–SIED-AIGO (28)]; five from North America [ACG 2013 and 2023 (29, 30), NASPGHAN (31) and ATA 2014 and 2016 (32, 33)]; two from South America [LATS (34) and SBEM (35)]; one International by WGO 2017 (36).

Analyzing conflicts of interest and funding, only 9 documents (45%) did not report any authors with conflicts of interest or funding to declare. In none of the publications, did the authors report receiving fees or financial support for writing the documents (see Supplementary File 1).

### 3.1 Celiac disease screening in patients with thyroid disease

**Figure 2:**
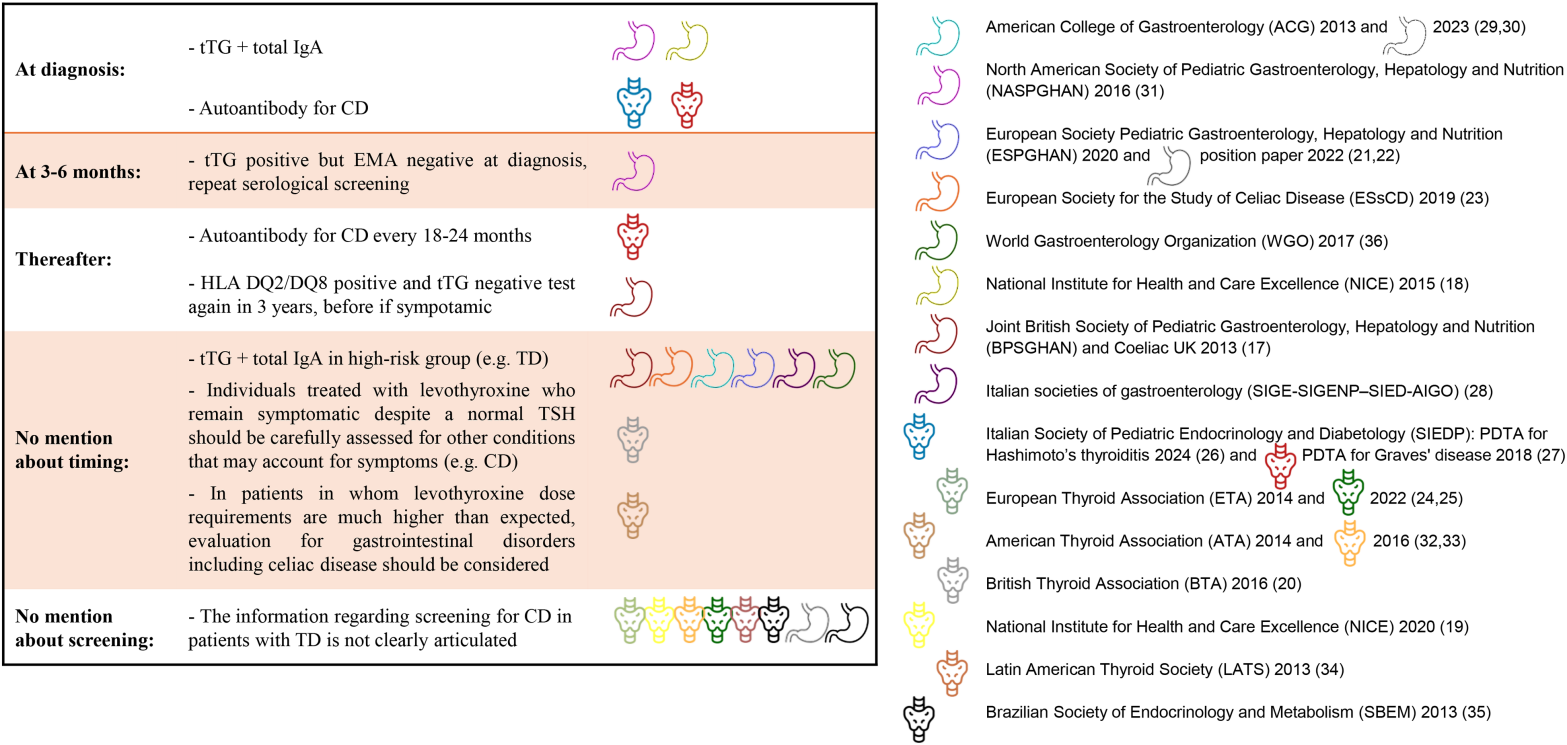
CD screening in newly diagnosed thyroid disease and during follow up, according to guidelines. tTG, Anti-transglutaminase antibodies IgA; CD, celiac disease; EMA, antiendomysium antibodies; HLA, human leukocytes antigen; IgA, Immunoglobulin A; TD, thyroid disease.

In patients with ATD, there is wide agreement among the guidelines to suggest anti-transglutaminase antibodies (tTG) immunoglobulin A (IgA) as the initial serological screening test for CD, complemented by a determination of total IgA levels to exclude IgA deficiency as shown in Figure 2. However, there is no consensus regarding timing for the screening. Special attention is given by certain guidelines to possible low-titer, false-positive, anti-tTG in patients with ATD. Human leukocyte antigen (HLA) genotyping is suggested only in a few documents.

Due to the prevalence of CD (4%–10%) in children and adolescents with autoimmune thyroiditis, the SIEDP (26) suggests performing CD screening at diagnosis of TD. Additionally in cases of co-occurrence of CD and ATD, autoimmune polyendocrine syndromes need to be ruled out (26, 37, 38). Based on ATA 2014 (32) and BTA (20) guidelines, evaluation for CD should also be considered in patients with hypothyroidism in whom levothyroxine dose requirements are much higher than expected, for BTA CD should also be assessed in patients with persistent symptoms despite normal serum TSH (20). In Italian societies of gastroenterology guideline, both HT and GD are in the list of situations in which testing for CD should be performed (28). Moreover, both American and European guidelines report that CD is more common in patients with underlying ATD (24, 32). However, no specific recommendation to screen for CD is provided in these guidelines.

The updated 2023 ACG guidelines (30) and WGO Global Guidelines for CD (36) recommend proactive case-finding screening for CD in patients with ATD, although they do not specify a time frame. NICE 2015 recommends offering serological testing for CD at diagnosis (18) of ATD. NICE 2020 proposes offering tests for thyroid dysfunction to adults, children, and young people with autoimmune diseases without making the converse recommendation or providing any other indications (19). The joint BSPGHAN/Coeliac UK guidelines (17), advise routine screening.

NASPGHAN 2016 (31) suggests testing all patients after the age of 3 years or at the time of initial diagnosis of the associated condition. The WGO 2017 guidelines (36) also recommend tTG IgA screening in patients with ATD as first-line tests for both symptomatic and asymptomatic patients with anti-endomysial antibodies (EMA) tests as confirmation. However, in both cases, if tTG IgA is negative at the first screening it does not specify after what period it is reasonable to repeat the serological test during follow up. Multiple biopsies of the duodenum (1 or 2 from bulb and 4 from distal duodenum) are necessary to confirm the CD diagnosis in cases of clinical or serological doubt. Both guidelines (31, 36) highlight the possibility of low-titer, false-positive anti-tTG in patients with ATD. The possibility of transient non-specific elevated levels of tTG, not indicative of CD, in patients with autoimmune disease, is also mentioned in other documents (23). A title of antibody <3 times the ULN should be viewed with suspicion. In these patients, based on expert opinion the NASPGHAN Clinical Report (31) suggests first obtaining an EMA-IgA test and only proceeding to biopsy if the EMA is positive. Moreover, it is not known what tTG-IgA level should be considered sufficient to recommend directly intestinal biopsy (39), therefore in these cases if the EMA is negative, and there are no other concerning symptoms, it may be acceptable to observe the patient and repeat the serological tests after 6–12 months. This approach is also suggested in the ESsCD Guidelines (23). Interestingly, there is no specific recommendation to avoid a biopsy sparing approach in these cases as specified by ESGPHAN for patients with T1DM.

In the ETA 2022 Guideline for the management of pediatric Graves' disease there is a comment about the risk of co - occurrence of GD and other autoimmune disorders such as T1DM and CD (25). Highlighting the same risk, the ISPED/SIEDP statement for Graves' disease suggests screening for commonly associated autoimmune diseases (27), while the ATA Guideline for hyperthyroidism does not mention CD (33). South American scientific societies do not provide specific recommendations for screening CD in patients with ATD (34, 35).

In BSPGHAN 2013 HLA-DQ2/DQ8 genotyping is recommended for asymptomatic children with ATD, if negative serology the optimum frequency for repeat blood testing is not specified, but every 3 years is considered reasonable if still asymptomatic (17). In the previous ESPGHAN 2012 guidelines (40) the screening for CD in patients with autoimmune disease known to be associated with CD may be started with HLA-DQ2/8, however this approach was not confirmed in the updated (2020) document (21), where HLA is no longer a mandatory test for diagnosis without biopsy, but the utility of CD screening in ATD is confirmed. Moreover, the ESPGHAN document underlines that in some at-risk groups, such as T1DM, HLA testing may not be cost-effective because of the high percentage of HLA positives within these groups (21). In the ESsCD Guideline (23) CD serology is indicated in HT and GD; HLA testing should be offered as the first step to asymptomatic patients with an increased risk of CD including ATD. If the patient is DQ2 and/or DQ8 positive, testing for CD (tTG-IgA test plus total IgA) should be repeated for at-risk children periodically. In WGO 2017 HLA DQ2/DQ8 is recommended in individuals with other autoimmune diseases or genetic disorders, who should be screened for CD including (36). In all guidelines where it is mentioned HLA test, the absence of DQ2/DQ8 haplotypes no further serological tests are needed.

In patients with selective total IgA-deficiency, tTG-IgA should be substituted with immunoglobulin G (IgG) based testing [tTG, antibodies to deamidated gliadin peptides (DGP) or EMA]. Several guidelines suggest using EMA-IgG and tTG-IgG at same time due to their lower specificity and sensitivity (18, 23, 28, 36). If there is a strong clinical suspicion of CD in an IgA-deficient patient, NASPGHAN guidelines suggest an intestinal biopsy even if all serological tests are negative (31). Similarly, a biopsy sparing approach in IgA deficient patients with positive IgG-based serological tests is not recommended in cases of value >10 ULN (21), and the recommendation for the follow-up of patients with IgA deficiency are scarce. An isolated positive IgG-based test with negative IgA-based tests in an IgA-competent individual is unlikely to be because of CD (31). The ACG 2013 (29) guidelines suggest a combination of different IgA and IgG antibodies in children younger than two years of age (for instance, anti- tTG IgA and DGP-IgG). The subsequent guidelines including ACG updated in 2023 (30) do not confirm this approach, as a combination of antibodies implies a higher sensitivity but reduced specificity, often leading to the necessity of overuse of histological confirmation.

### 3.2 Thyroid screening in patients with celiac disease

**Figure 3:**
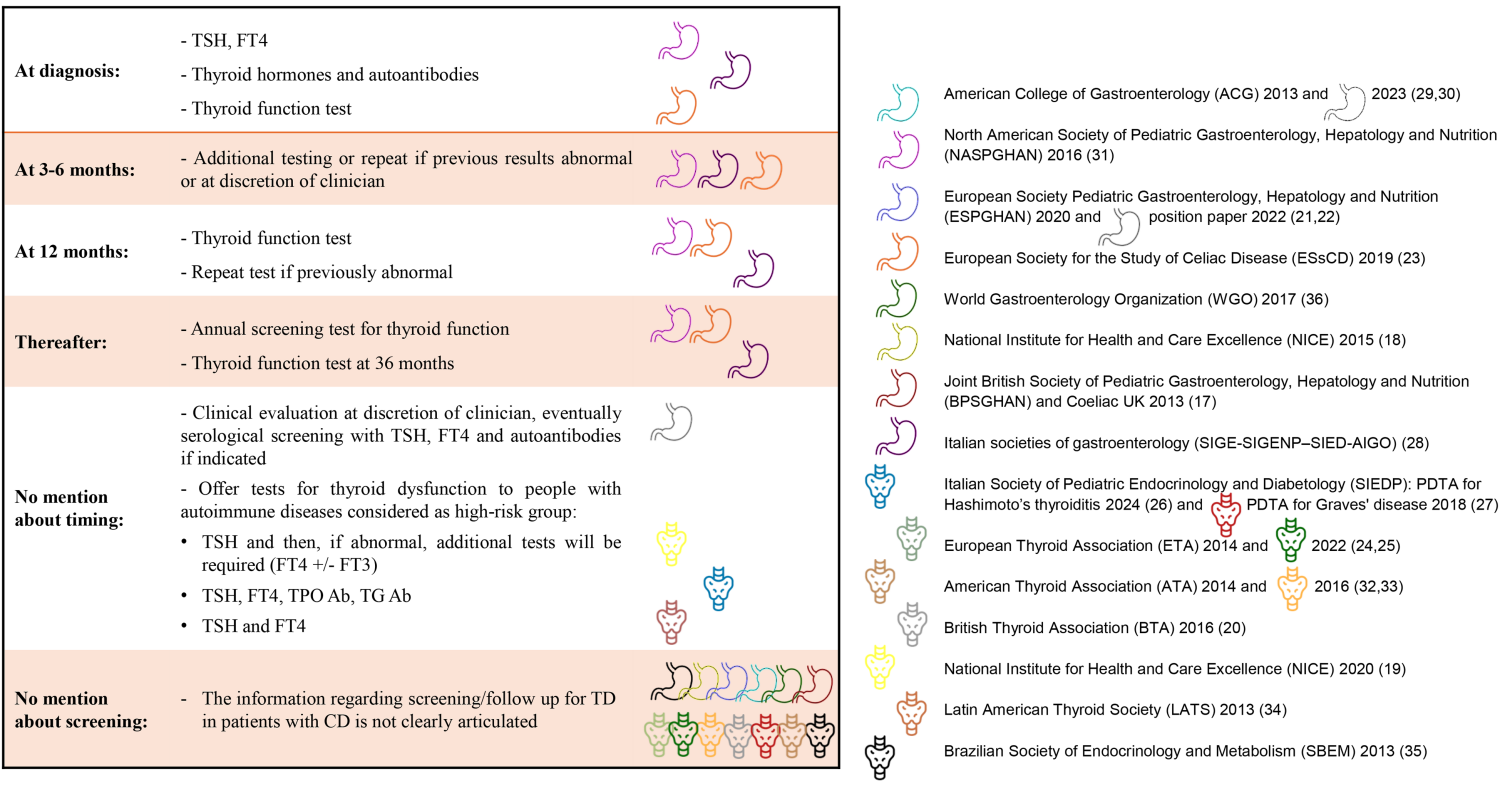
Tyroid function screening in newly diagnosed celiac disease and during follow up, according to guidelines. TSH, thyroid stimulating hormone; FT3, free triiodothyronine; FT4, free thyroxine; TPO Ab, anti-thyroid autoantibodies; TG Ab, thyroglobulin antibodies.

Despite the widespread agreement on the utility of screening for CD in patients with ATD, the converse recommendation has not been clearly expressed in the guidelines examined, as shown in Figure 3. The TSH is the recommended test in all cases of thyroid function screening, in several documents it is recommended to combine *free thyroxine* **(**FT4) in the same sample. Autoimmunity testing and *free triiodothyronine* (FT3) is recommended in cases of clinical suspicion of ATD.

In the follow up assessment by a clinician and dietitian the joint BSPGHAN/Coeliac UK guidelines suggest including a review of symptoms, growth, physical examination, micronutrient intake and adherence to life-long gluten-free diet (GFD) during the follow-up of patients with CD, however there is no mention of thyroid serological test (17). Similar approaches are found in other guidelines. The ACG guidelines updated in 2023 propose an active surveillance of comorbidities (especially coexistent autoimmune diseases) in patients with CD, through a clinical follow up, without however including thyroid function in the blood tests suggested at diagnosis or follow-up (30). NICE 2015 guidelines suggest offering an annual review to people with CD to measure weight/height and review symptoms, referring the patients to their general practitioner if concerns are raised (in the annual review). The general practitioner or consultant should assess the need for specific blood tests, however there is no advice about which test or timing (18). On the other hand, the NICE 2020 guidance for TD assessment and management, recommends offering tests for thyroid dysfunction to children and adults with autoimmune diseases measuring only TSH, testing in the same sample FT4 or both FT4 and FT3 in cases where TSH level suggests, respectively, hypothyroidism or hyperthyroidism (19).

ESPGHAN Guidelines for Diagnosing CD updated in 2020 recommend considering testing for CD in children and adolescents with specific predisposition conditions including TD (21). The 2022 position paper on management and follow-up confirms that children with CD have an elevated risk of ATD, however it underlines the absence of evidence to support a recommendation for assessment of FT4 or TSH blood levels during follow up of CD. Therefore, serological screening for thyroid function (or autoantibodies) is not recommended (91% Agreement), but only to be considered by the clinician during follow-up of children with CD after clinical evaluation (22).

In the document edited by NASPGHAN a specific recommendation is provided to test the thyroid function, measuring TSH and FT4, at diagnosis of CD and annually after symptom resolution and normalization of CD serology. It is emphasized that this has been developed on a consensus of expert opinion (31). A similar approach is presented also in the ESsCD Guideline (23), thyroid function tests are suggested at diagnosis, at 6 months if abnormal at diagnosis, yearly for up to 36 months and thereafter every 1–2 years. Notably, the thyroid function tests (it is not indicated which tests) are the only routine test suggested after 12 months (together with CD serology). However, the suggested follow-up scheme is applied only to adult CD patients without specific recommendations for the follow up in pediatric patients. In the guidelines of the Italian societies of gastroenterology, at each follow-up medical consultation, the patient's clinical condition should be reviewed and compared with the clinical picture at diagnosis, repeated blood tests are suggested during the first 3 years only if altered at diagnosis, while thyroid function test is recommended at 36 months from CD diagnosis (28).

The ISPED/SIEDP suggests measuring TSH, FT4, FT3, anti TPO and anti TG in case of autoimmune disease, however without specifying when to test (26). LATS recommend testing for thyroid function (measuring TSH, fT3 and FT4) in people with personal history of autoimmune disease, underlining a special attention to Vitiligo, Sjögren's syndrome, Systemic Lupus Erythematosus, Alopecia and Rheumatoid Arthritis (34).

The guidelines of various endocrinology societies do not provide specific recommendations for the risk management of ATD in patients with CD (19, 24, 25, 27, 32, 33, 35).

## 4 Discussion

This study aims to systematically review and compare international guidelines from endocrinological and gastroenterological pediatric societies over the past decade concerning the screening for TD in children with CD and vice versa. Our findings reveal a lack of consistency among these recommendations and underscore the need for a more unified approach. Notably, most guidelines recommend routine screening for CD in patients with ATD, while the recommendation for routine screening for TD in patients with CD is not clearly stated.

Autoimmune thyroid disease is the most common autoimmune condition associated with CD. Patients with CD or ATD have a higher prevalence of other autoimmune diseases, including T1DM, Addison's disease, vitiligo, alopecia, hypogonadism, chronic autoimmune gastritis and systemic lupus erythematous (41–43). Given the risk and the potential complications associated with untreated CD, there is a general agreement among guidelines recommending screening for CD in asymptomatic patients at the time of ATD diagnosis. Previous ACG and ESPGHAN guidelines suggest screening with a combination of tTG-IgA and DGP-IgG in children younger than two years of age (29, 40). Recent guidelines are concordant and suggest anti-tTG-IgA and total IgA as the initial serological tests. If IgA deficiency is identified, both DGP-IgG and tTG-IgG should be tested. Esophagogastroduodenoscopy with multiple biopsies of the bulb and distal duodenal remains the gold standard for diagnosing suspected CD. The ESPGHAN 2012 guidelines endorsed the possibility of omitting duodenal biopsy in children with suspected CD for the first time (40). This possibility was limited to specific conditions: presence of classic symptoms, high tTG-IgA (>10 times the upper limit of normal), EMA-IgA positivity, and presence of HLA DQ2 or DQ8. This approach was subsequently adopted by a plurality of international guidelines (18, 23, 36). The last ESPGHAN and ACG guidelines indicate that high tTG-IgA level and a positive EMA test from a second blood sample is sufficient to diagnose CD also in asymptomatic children and adolescents, removing HLA and symptoms as crucial criteria for a biopsy sparing approach (21, 30). This diagnosis approach should involve shared decision-making with the family (21, 30).

Few guidelines address the lower specificity of tTG screening for CD in patients presenting with new autoimmune diseases, such as ATD. This issue has been extensively studied in T1DM, where tTG levels can fluctuate and may even normalize spontaneously in about 50% of the patients who continue to consume gluten (44, 45). Recent evidence suggests (44) that the cut off levels greater than three times the upper limit of normal or a positive EMA test are less likely to normalize spontaneously and thus necessitate biopsy confirmation. In asymptomatic cases with low tTG titers, careful serological follow-up is essential, and the timing of biopsies may be deferred in agreement with the family (46). All cases of isolated low tTG titers with a negative EMA test, where families opt against endoscopic biopsy, should be monitored serologically for at least five years, with this data recorded as a clinical risk and an opportunistic screening suggested for the future.

Patients with T1DM share a common genetic risk with CD, which makes HLA typing an ineffective and costly screening method for CD in these individuals (47). The cost-effectiveness and utility of HLA testing in ATD should be further evaluated in ongoing population screening studies (48).

While there is general consensus on the high comorbidity between CD and ATD, inconsistencies exist in the screening protocols. There is no standardized agreement on the frequency or method of routine screening for ATD in children with CD over time. It remains unclear whether screening for TD in this specific population meets the criteria for population screening, which tests or combination of tests (such as thyroid function test and/or autoantibody tests) are most appropriate, and when to re-test if initial results are negative. The most relevant paediatric endocrine society in Europe, the European Society for Paediatric Endocrinology (ESPE), it has not yet endorsed guidelines on ATD.

A meta-analysis involving 6,024 patients with ATD found that approximately 1 in 62 had biopsy-verified CD. The prevalence was higher in children [6.2% (CI 4.0%–8.4%)] and in patients with hyperthyroidism [2.6% (CI 0.7%–4.4%)] than those with hypothyroidism [1.4% (CI 1.0%–1.9%)] (5). Nevertheless, few guidelines clearly address the management of thyroid function follow- up in patients with CD. Furthermore, the 2022 ESPGHAN position paper recommend testing for TSH, FT4 and autoantibodies only if indicated by clinical evaluation (22).

Thyroid dysfunction can often be asymptomatic or paucisymptomatic for many years, increasing the risk of late diagnosis, as reported by various screening studies (3, 49, 50). The effectiveness of screening strategies to improve the diagnosis rates of CD or ATD in these patient populations remains limited. For instance, Italian CD screening studies show that a passive case- finding detects only about one-third of CD cases in the general population, while an active case-finding strategy still misses a quarter of cases (51).

In the TRIAD study, only six of the 99 (6.1%) children with ATD autoantibodies had elevated TSH levels and signs of thyroiditis on ultrasound, all without any clinical symptoms of ATD (3).

From a clinical perspective, optimizing the diagnostic approach for these potentially coexisting autoimmune diseases is essential to identify patients who could benefit from early treatment. Unrecognized TD in pediatric patients with CD can lead to significant harm, including growth impairment, metabolic disturbances, chronic fatigue and low quality of life. Conversely, undiagnosed CD in patients with ATD can result in malabsorption, nutrient deficiencies, long-term gastrointestinal complications, and an increased risk of other autoimmune disorders.

Our analysis indicates that a balanced approach is necessary, weighing the risk of excessive blood testing against the potential for missing a diagnosis of CD or ATD. Each case should be carefully assessed, taking into account the overall risk associated with various factors such as family history, sex, other autoimmune disorders, and age.

**Figure 4:**
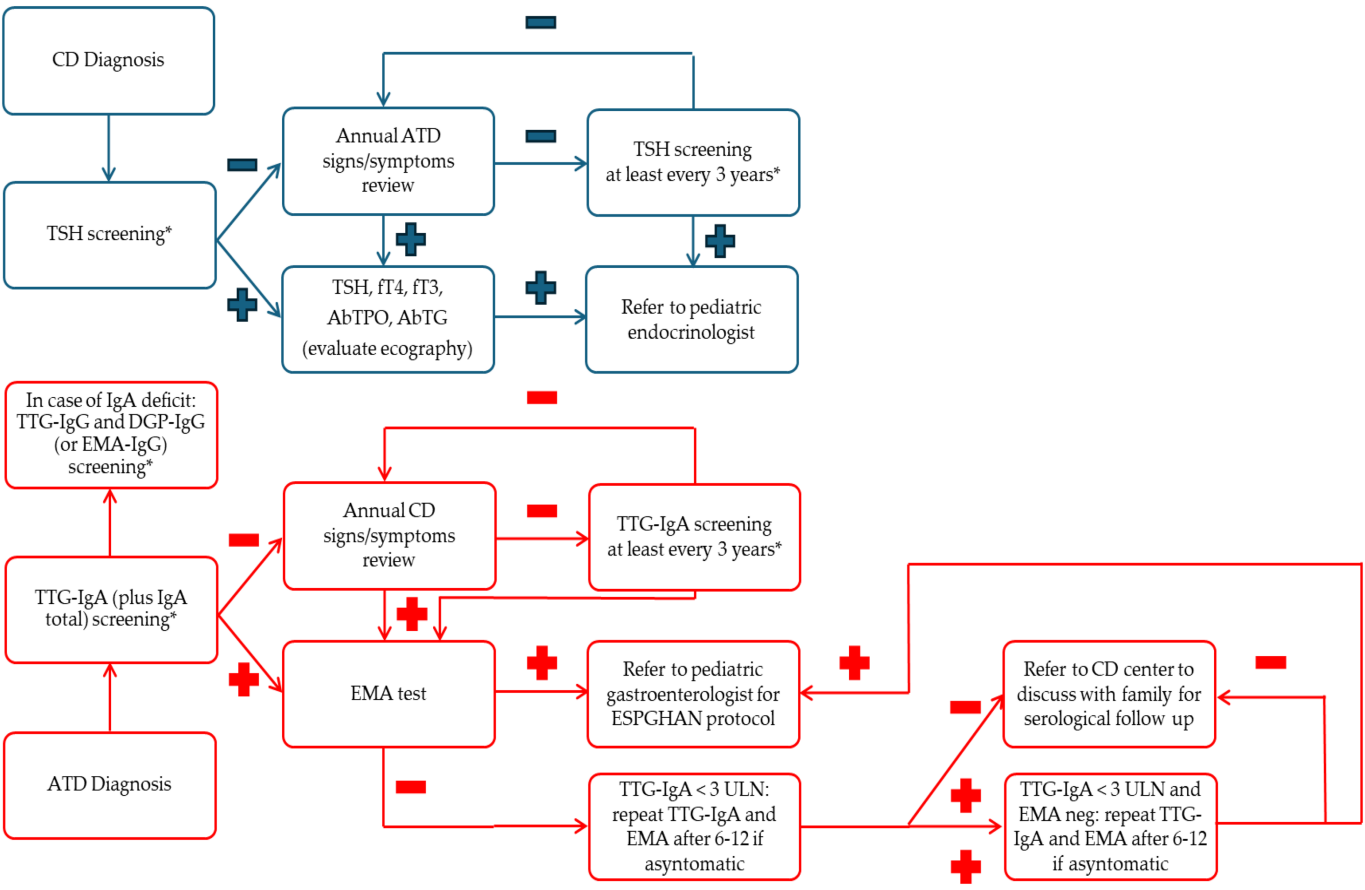
Practical flow charts. * Considered opportunistic screening; −: normal value; +: altered value; ATD, autoimmune thyroid disease; CD, celiac disease; DGP, anti-deamidated gliadin peptide antibodies; EMA, anti-endomysium antibodies; ESPGHAN, European Society Pediatric Gastroenterology, Hepatology and Nutrition; FT3, free triiodothyronine; FT4, free thyroxine; IgA, immunoglobulin A; IgG, immunoglobulin G; Neg, negative; Pos, positive; TG Ab, thyroglobulin antibodies; TPO Ab, anti-thyroid autoantibodies; TSH, thyroid stimulating hormone; TTG, anti-transglutaminase antibodies; ULN, upper limit normal.

Based on the data presented by this systematic review and the extensive evidence reported in the guidelines, the following conclusions are proposed as represented in the practical flow charts (Figure 4).

1. Screening for coexisting conditions: all patients with CD should be screened for ATD at onset, and vice versa.
2. Regular follow-up: tTG IgA testing for children with ATD, TSH and FT4 testing for those with CD should be included in routine follow-up (at least every three years) or opportunistic screening (such as patients undergoing routine blood test). Patients must follow a gluten-containing diet: a minimum of 10 grams of gluten per day for 12 weeks (52). Particular attention must be paid to patients with IgA deficiency due to the risk of autoimmune diseases and lower reliability of the tTG-IgG test.
3. Monitoring symptoms: The appearance of signs or symptoms consistent with CD or ATD should trigger investigation at every follow-up visit (at least annually), and if there is any uncertainty, serological screening must be repeated.
4. Targeted testing: FT3, thyroid peroxidase antibodies, and TG tests for ATD, as well as EMA for CD, should be reserved for cases with a first positive screening result.
5. Management of low tTG levels: In asymptomatic patients with fluctuating isolated low tTG IgA levels (i.e., <3 times the upper limit of normal) who opt not to undergo endoscopic biopsy, referral to a specialized center for CD is necessary. These patients should be monitored for at least five years with repeat serological testing to confirm normalization of their results. An increase in tTG titers, EMA seroconversion, or the emergence of symptoms consistent with CD should prompt a biopsy.

This study has some limitations. The database search was limited to PubMed, focusing only on publications from the last decade. Additionally the exclusion of non-English guidelines may have resulted in the omission of important recommendations from other scientific societies. The variability in guideline formats and terminologies also posed challenges for direct comparison. While our flow charts, summarizing 20 guidelines from various regions and organizations, serve as useful tools for clinical practice, prospective studies are needed to establish a uniform approach based on solid evidence and cost- effectiveness.

## 5 Conclusions

In conclusion, while significant progress has been made in understanding the relationship between CD and TD, there is still a pressing need for harmonized guidelines. A collaborative effort among international pediatric gastroenterological and endocrinological societies could pave the way for more uniform and effective management of these frequently associated autoimmune diseases.

Future research should address existing knowledge gaps to develop standardized, evidence-based screening protocols for CD and TD in pediatric patients, including considerations on who to screen and when, as well as the role of HLA testing. Ongoing longitudinal studies assessing the outcomes of different screening strategies could provide valuable insights into the most effective approaches, helping to balance the overall “cost” of screening these at-risk populations against the potential consequences of missed diagnoses.

## Data Availability

The raw data supporting the conclusions of this article will be made available by the authors, without undue reservation.
