# Supplementary material for: A systematic review of guidelines on screening for celiac disease in children with thyroid disease and vice versa
Source: Front Pediatr. 2025 Mar 31;13:1538409. doi: 10.3389/fped.2025.1538409 (PMC11994688; doi:10.3389/fped.2025.1538409)
Supplement: Supplementary file 1 [file Supplementaryfile1.docx]

Conflicts of interest and funding statements:

1. SIGE, AIGO, SIED, SIGENP 2022: no conflicts of interest, no funding.
2. BSPGHAN 2013: no conflicts of interest
3. NICE 2015: The authors declaration of interest can be found in ttps://www.ncbi.nlm.nih.gov/books/NBK343375/
4. NASPGHAN 2016: no conflicts of interest
5. WGO 2017: no conflicts of interest
6. ESPGHAN 2020: All guideline members’ conflicts of interest have been noted and registered on the ESPGHAN website. The guideline was funded by ESPGHAN and was developed in collaboration with AOECS.
7. ESPGHAN position paper 2022: Mearin received receipt of grants/research supports from Research Grant Eurospital, Thermofisher and BioHit and served as member of advisory board and consultancy an speaker from Thermofisher. Agardh received receipt of grants/research supports from Novo Nordisk Foundation and served as member of advisory board from Takeda and Thermofisher. Anunes received receipt of payment/honorarium for lectures from Danone, Catassi received receipt of payment/honorariumm for consultation from Dr Schar Food. Dolinsek received receipt of payment/honorarium for lectures from Medis, Abbot, Merit. Guandalini served as member of advisory board from ExeGIPharma, Imaware. Koletzko receved receipt of grants/research supports from Mead Johnson, Biogaia; served as member of advisory board from Abbvie, Danone, Jannsen, Sanofi, Takeda; receipt of payment/honorarium for lectures from Danone, Mead-Johnson, Nestlè Nutrition, Pfizer, Takeda; receipt of payment/honorarium for consultation from Danone. Korponay-Szabò other support from patent on celiac disease repid test lcensed to Labsystems Oy, Vantaa, Finland. Kurppa received receipt of grants/researc supports from Finnish Pediatric Foundation, Paivikki, and Sakari Sohlberg Foundation;served as member of advisory board from Finnish Coealich society; receipt of payment/honorarium for consultation from Takeda. Rodriguez-Herrera received other support from Patent and detecting gluten peptides in human fluids, March 29, 2019-Universidad de Sevilla. Shamir received receipt of grants/research supports from Hemsley Foundation; served as member of advisory board: Nestle Nutrition Institute, Teva; receipt of payment/honorarim forlectures from Abbott, Nutricia, Nestle Nutrition Institute; receipt of payment/honorarium for consultation from Abbott, Else, Nutricia, Nestle Nutrition Institute and Stock shareholder from NGS. Ciacci received receipt of honoraria or consultation fees from Takeda, GSK, Mediserve, Biogen Celltrion. The remaining authors report no conflicts of interest. The guideline was funded by ESPGHAN and was developed in collaboration with AOECS.
8. ESSCD 2019: Professor Sanders has received educational research grants from Dr Schaer (a gluten-free food manufacturer) and Tillotts Pharma (distributor of a POCT for CD) for investigator-led studies. Dr Schaer and Tillott’s Pharma did not have any input in the study design, access to study data, interpretation of the findings or drafting of the manuscript. Professor Lundin: KEAL has been involved in clinical trials conducted by Alvine Pharmaceutical, Cellimune and Dr. Falk Pharma. The other authors declare that there is no conflict of interest
9. ACG 2023: no conflicts of interest. No financial support
10. ACG 2013 : Financial support: the authors are supported by National Institutes of Health DK-57982 (J.A.M.), 1K08 DK090150 (A.H.C.), and American College of Gastroenterology Junior Faculty Development Award (A.R.-T.). Potential competing interests: Dr Rubio-Tapia and Dr Calderwood have nothing to declare. Dr Hill serves on the editorial boards of the *Journal of Pediatrics* and *Journal of Pediatric Gastroenterology and Nutrition* . Dr Kelly acts or has acted as a scientifi c and medical advisor to Alba, Alvine, and ImmunosanT and has received research funding support on CD from Alba and Shire. Dr Murray has received grant support from Alba Th erapeutics ( > $ 50,000), served on the Advisory Board of Alvine Pharmaceuticals ( < $ 10,000), and served as consultant to Ironwood ( < $ 10,000), Flamentera ( < $ 10,000), Actogenix ( < $ 10,000), Bayer Healthcare Pharmaceuticals ( < $ 10,000), Vysera Biomedical ( < $ 10,000), 2G Pharma ( < $ 10,000), ImmunosanT ( < $ 10,000), and ShireUS ( < $ 10,000).
11. ETA 2022: Declaration of interest: Simon Pearce received speaker fees from Merck, Sanofi, Berlin Chemie, Quidel and is member of the clinical advisory board of Apitope. The other authors have nothing to disclose. Funding : the Department of Anja Eckstein received funding from Horizon Therapeutics plc to conduct clinical studies on the use of Teprotumamab in GO. No financial support was received writing this guideline.
12. ATA 2016: Julie Ann Sosa discloses significant financial interests or other relationships as a Member, Data Monitoring Committee for Medullary Thyroid Cancer Registry supported by Novo Nordisk, AstraZeneca, GlaxoSmithKline, and Eli Lilly. The remaining authors disclose no significant financial interests or other relationships with commercial interests.
13. NICE 2020: Competing interests: We declare the following interests based on NICE's policy on declaration of interests (available at https://www.nice.org.uk/about/who-we-are/ policies-and-procedures): MV and JG have no interests to declare, and the other authors’ full statements can be viewed at https://www.nice.org.uk/guidance/ng145/ documents/register-of-interests. Funding: MV and JG were both employees of the National Guideline Centre at the time of development of the guideline discussed in this summary. The National Guideline Centre was commissioned and funded by NICE to develop this guideline. No authors received specific funding to write this summary
14. BTA 2016: Competing interest: Onyebuchi Okosieme is conducting research into the genetics of thyroid hormone replacement (GENTHYR Study) funded by Concordia International (formerly AMco), manufacturers of levothyroxine (T4) and liothyronine (T3). Onyebuchi Okosieme and Mark Vanderpump are executives of the British Thyroid Association (BTA) and are co-authors of the BTA statement on hypothyroidism
15. SIEDP Hashimoto: no reference about conflicts of interest and funding
16. SIEDP Graves: no reference about conflicts of interest and funding
17. ATA 2014: no funds were received from commercial sources for the development of this document. Task force members did not receive any funding or gifts for their participation and paid for their own travel expenses and registration related to face-to-face meetings. Possible conflicts of interest of potential task force members were reviewed by the officers of the ATA. No confirmed task force member was considered to have a serious conflict that precluded inclusion on the task force
18. ETA 2014: The task force had no commercial support, and the members declared no conflict of interest.
19. LATS 2013: no potential conflict of interest relevant to this article was reported
20. SBEM: no potential conflict of interest relevant to this article was reported.
